# *Fusarium incarnatum*: a paradigm for One Health pathogen dynamics across humans, animals, and the environment

**DOI:** 10.1128/spectrum.01919-25

**Published:** 2025-11-26

**Authors:** Ahmed Namisy, Ying-Chen Wu, Kuo-Hsi Lin, Wei-Che Hsu, Wen-Hsin Chung

**Affiliations:** 1 Department of Plant Pathology, National Chung Hsing University 34916, Taichung, Taiwan; 2 Research Center for Animal Medicine, National Chung Hsing University 34916, Taichung, Taiwan; 3 Graduate Institute of Veterinary Pathobiology, College of Veterinary Medicine, National Chung Hsing University 200384, Taichung, Taiwan; 4 Tungs' Taichung MetroHarbor Hospital, Taichung, Taiwan; 5 Department of Post-Baccalaureate Medicine, National Chung Hsing University 34916, Taichung, Taiwan; 6 Master Program for Plant Medicine and Agricultural Practice, National Chung Hsing University 34916, Taichung, Taiwan; Agricultural Research Organization Volcani Center, Rishon LeZion, Israel

**Keywords:** *Fusarium incarnatum*, One Health, cross-infection

## Abstract

The *Fusarium incarnatum-equiseti* species complex (FIESC) is a harmful plant pathogen responsible for diseases, such as root rot, fruit rot, leaf blight, and wilt, in various crops. Recently, it has been increasingly associated with both superficial and invasive infections in humans and animals. Additionally, this pathogen produces mycotoxins, posing significant risks to public health. This study aims to characterize the molecular and morphological characteristics of FIESC isolates obtained from various sources, including human, veterinary, and plant samples. Molecular identification was performed using multilocus sequence typing (MLST), targeting the internal transcribed spacer (ITS) rDNA, the second-largest subunit of the RNA polymerase gene (RPB2), and translation elongation factor 1 alpha (TEF-1α). Based on MLST analysis, all FIESC isolates were found to belong to the Incarnatum clade and were classified into two species: *F. irregulare* and *F. pernambucanum*. Furthermore, these isolates demonstrated the ability to penetrate and colonize human nails, porcine skin, and muskmelon fruits. In this study, we report members of FIESC as a newly identified pathogens in Taiwan capable of cross-infection among humans, animals, and plants. These findings highlight the dual significance of *Fusarium*: it poses a serious threat to agriculture by compromising plant health and crop yields while also representing a public health risk due to its ability to infect humans and animals.

**IMPORTANCE:**

The *Fusarium incarnatum-equiseti* species complex (FIESC) is a significant plant pathogen affecting a wide range of crops, leading to substantial agricultural losses worldwide. In this study, we report that *Fusarium* isolates belonging to *F. pernambucanum* and *F*. *irregulare* obtained from various sources, including respiratory specimens from human, cat, rabbit, and turtle, along with plant samples from muskmelon fruits, represent newly identified pathogens in Taiwan. Our findings suggest that these isolates possess the potential for cross-infection among plants, animals, and humans, underscoring their importance in both agricultural and medical contexts. Notably, the plant isolates PLMF1 and BDMF7 demonstrate the ability to transition from plant pathogens to zoonotic pathogens, highlighting a concerning adaptability.

## INTRODUCTION

The fungal genus *Fusarium* is one of the most significant groups of plant-pathogenic fungi, affecting a wide range of crops worldwide (1). These fungi are soil-borne diseases and can persist for long periods in the soil, surviving on crop residues and the roots of non-host plants as chlamydospores (2). *Fusarium* is widespread due to its ability to grow on various substrates and effective dispersal mechanisms (3). Additionally, *Fusarium* species have been identified in the water distribution systems of hospitals and residences, where they can survive for years (4). Currently, *Fusarium* is ranked among the 10 most destructive fungal plant pathogens (5). With a broad host range, *Fusarium* spp. can cause various diseases in many plant species. The pathogen infects certain parts of plants, such as grains, seedlings, heads, roots, and stems, leading to various diseases that reduce product quality and diminish crop yields (6). The genus *Fusarium* comprises at least 300 species grouped into approximately 23 species complexes based on multi-locus phylogenetic analyses (7). *Fusarium* species complexes, associated with human and animal infections, include the *F. incarnatum-equiseti* species complex (FIESC), *F. tricinctum* species complex (FTSC), *F. sambucinum* species complex (FSAMSC), *F. solani* species complex (FSSC), *F. fujikuroi* species complex (FFSC), *F. oxysporum* species complex (FOSC), *F. dimerum* species complex (FDSC), and *F. chlamydosporum* species complex (FCSC) (8).

In humans, *Fusarium* species is recognized as the second most common mold infection after *Aspergillus* (8). The most common infections caused by *Fusarium* are superficial, localized diseases such as keratitis and onychomycosis in immunocompetent patients (9). However, in severely immunocompromised patients, *Fusarium* can cause locally invasive or disseminated infections (9), particularly in those who have undergone allogeneic hematopoietic stem cell transplantation (HSCT) due to significant immunosuppression and prolonged neutropenia (8). Invasive disease caused by *Fusarium* spp. includes cutaneous infection, chronic rhinosinusitis, pneumonia, brain abscess, and fatal fungemia (10). *Fusarium* species commonly cause opportunistic infections in animals, including seahorses, turtles, dolphins, dogs, horses, and pigs (6, 11). It is responsible for various animal mycotic infections, such as keratitis and dermatitis (12). In addition, *Fusarium* spp. have been reported to cause systemic infection in goats and dogs, including meningoencephalitis, chronic ulcerative dermatitis, and lesions in the kidneys, abdominal lymph nodes, and lungs (11, 13, 14).

Besides the direct infection of plants, humans, and animals, *Fusarium* spp. can also produce mycotoxins, including trichothecenes and zearalenone (15, 16). *Fusarium* mycotoxins can infiltrate the food chain through contamination during crop production and food processing, ultimately posing a threat to human and animal health (17). These mycotoxins contribute to worldwide losses amounting to millions of dollars annually due to their impact on human health, animal health, and condemned agricultural products (16). The most significant effect of *Fusarium* species on livestock arises from feed contaminated with mycotoxins (18). Poultry, pigs, and aquatic vertebrates are highly sensitive to mycotoxins due to their high consumption rates and chronic exposure to cereal mycotoxins (18).

Currently, the cross-kingdom infection of *Fusarium* spp. in plants and humans has been reported in previous studies. *Fusarium* spp. isolated from keratitis patients retain their infective capacity during the cross-kingdom reinfection transmission from humans to plants (19). For example, members of *F. solani* are host-specific pathogens with an extensive host range of agriculturally important crops, such as potato, tomato, pepper, and eggplant (20). However, they are also increasingly recognized as causative agents of human and animal mycoses (21). Moreover, FIESC has been reported to cause infections in humans, animals, and plants (12, 22, 23). These cross-infections pose serious threats to food safety and public health. In this study, FIESC was successfully isolated from various sources, including human, veterinary, and agricultural environments in Taiwan. These isolates were analyzed phylogenetically using multilocus sequence typing (MLST), targeting the internal transcribed spacer (ITS) rDNA, the second-largest subunit of the RNA polymerase gene (RPB2), and translation elongation factor 1 alpha (TEF-1α). Additionally, we investigated the potential for cross-infection of these isolates in plants, human nails, and porcine skin.

## MATERIALS AND METHODS

### Pathogen isolates

This study analyzed six *Fusarium* isolates belonging to FIESC that were obtained from human, veterinary, and plant sources (Table 1). The isolate FS10 was recovered from a sputum sample of a patient diagnosed with fungal pneumonia at Tungs’ Taichung MetroHarbor Hospital, Taichung, Taiwan. The patient had a history of liver transplantation, and after antifungal treatment, the symptoms resolved. Veterinary isolates LD1, FS097, and FS405 were recovered from three different animal species, all presenting with respiratory clinical signs, at the clinical microbiology laboratory of the Research Center for Animal Medicine in Taichung, Taiwan. Specifically, isolate LD1 was cultured from a cat’s lung via bronchoalveolar lavage, isolate FS097 from a rabbit’s purulent nasal discharge, and isolate FS405 from a turtle’s (*Chelonoidis denticulata*) tracheal discharge via tracheal wash. Two plant isolates, PLMF1 and BDMF7, obtained from rotted melon fruits, were generously provided by the Plant Parasites and Pesticide Resistance Molecular Diagnosis Laboratory (PRMD), National Chung Hsing University, Taichung, Taiwan. The PRMD lab previously identified PLMF1 and BDMF7 as *Fusarium incarnatum-equiseti* species complex, based on their morphological and molecular characteristics.

**TABLE 1:** *Fusarium incarnatum-equiseti* species complex isolates used in phylogenetic analysis

				GenBank accession		
*Fusarium* species	Isolate	Host	Origin	ITS	TEF 1-α	RPB2
*F. irregulare*	FS097	Rabbit nose	Taiwan	PV104033.1	PV368517.1	PV368518.1
	LD1	Cat lung	Taiwan	PV076706.1	LC863198.1	PV523833.1
	LC7188	Bamboo	China	MK280829.1	MK289629.1	MK289783.1
	NRRL 34001	Human foot wound	Texas	GQ505714.1	GQ505625.1	GQ505803.1
	PLMF1	Melon	Taiwan	PP092085.1	LC802793.1	LC810989.1
*F. pernabucanum*	FS405	Turtles	Taiwan	PV103224.1	LC864520.1	PV472292.1
	FS10	Human sputum	Taiwan	PV104032.1	LC864519.1	LC863179.1
	NRRL 32864	Human	Texas	GQ505702.1	GQ505613.1	GQ505791.1
	NRRL 36548	Banana	Congo	GQ505744.1	GQ505655.1	GQ505833.1
	NRRL 34070	Tortoise	Illinois	GQ505731.1	GQ505642.1	GQ505820.1
	BDMF7	melon	Taiwan	PP087967.1	LC800433.1	LC804558.1
*F. sulawesiense*	NRRL 34056	Human bronchial wash	Illinois	GQ505729.1	GQ505640.1	GQ505818.1
	NRRL 43730	Contact lens	Mississippi	GQ505758.1	GQ505669.1	GQ505847.1
	NRRL 34059	Human blood	Illinois	GQ505730.1	GQ505641.1	GQ505819.1
*F. luffae*	NRRL 31167	Human sputum	Texas	GQ505697.1	GQ505608.1	GQ505786.1
	LC12167	Luffa	China	MK280807.1	MK289601.1	MK289754.1
*F. caatingaense*	NRRL 36575	Juniperus chinensis leaf	Hawaii	GQ505745.1	GQ505656.1	GQ505834.1
	NRRL 34003	Human sputum	Texas	GQ505716.1	GQ505627.1	GQ505805.1
*F. tanahbumbuense*	NRRL 43297	Spartina rhizomes	Connecticut	GQ505746.1	GQ505657.1	GQ505835.1
	NRRL 34005	Human intravitreal fluid	Minnesota	GQ505718.1	GQ505629.1	GQ505807.1
*F. incarnatum*	NRRL 32867	Human	Texas	GQ505705.1	GQ505616.1	GQ505794.1
	NRRL 13379	*Oryza sativa*	India	GQ505680.1	GQ505591.1	GQ505769.1
*F. nanum*	LC12168	*Musa acuminata*	China	MK280794.1	MK289602.1	MK289755.1
	LC1385	Saudi Arabia	Tomato	MK280781.1	MK289612.1	MK289765.1
*F. croceum*	NRRL 3214	Unknown	Unknown	GQ505676.1	GQ505587.1	GQ505765.1
	NRRL 3020	Unknown	Unknown	GQ505675.1	GQ505586.1	GQ505764.1
*F. equiseti*	NRRL 36136	Unknown	Unknown	GQ505733.1	GQ505644.1	GQ505822.1
	NRRL 26419	Soil	Germany	GQ505688.1	GQ505599.1	GQ505777.1
*F. clavum*	NRRL 34032	Human abscess	Texas	GQ505724.1	GQ505635.1	GQ505813.1
	NRRL 32871	Human abscess	Texas	GQ505708.1	GQ505619.1	GQ505797.1
*F. flagelliforme*	NRRL 31011	*Thuja*sp.	Germany	GQ505695.1	GQ505606.1	GQ505784.1
	NRRL 26921	Wheat	Germany	GQ505689.1	GQ505600.1	GQ505778.1
*F. brevicaudatum*	NRRL 43638	Manatee	Florida	GQ505754.1	GQ505665.1	GQ505843.1
	NRRL 43694	Human eye	Texas	GQ505757.1	GQ505668.1	GQ505846.1
*F. duofalcatisporum*	NRRL 36448	Phaseolus vulgaris seed	Sudan	GQ505741.1	GQ505652.1	GQ505830.1
	NRRL 36401	Cotton	Mozambique	GQ505740.1	GQ505651.1	GQ505829.1
*F. compactum*	NRRL 36323	Cotton yarn	England	GQ505737.1	GQ505648.1	GQ505826.1
	NRRL 28029	Human eye	California	GQ505691.1	GQ505602.1	GQ505780.1
*F. concolor*	NRRL 13459	Plant debris	South Africa	PP336538.1	GQ505674.1	GQ505852.1

### Phylogenetic analysis

The genomic DNA was extracted from mycelium grown on PDA medium for one week using the DNeasy Blood and Tissue Kit (QIAGEN GmbH, Hilden, Germany). The internal transcribed spacer (ITS) rDNA, translation elongation factor 1-α (TEF 1-α) gene, and RNA polymerase II second largest subunit (RPB2) gene were PCR amplified and sequenced using primers of ITS1/ITS4 (24), EF1/EF2 (25), and 5F2/7CR (26), respectively. The PCR was performed in a 25 µL reaction volume, consisting of 0.5 µL (10 µmol/L) of each forward and reverse primer, 5 µL of PCR Master Mix (BioKit, Taiwan), 18 µL of nuclease-free water, and 1 µL of DNA template. PCR amplifications were conducted using a Labcycler 48 (SensoQuest GmbH, Göttingen, Germany) under the conditions described by White et al. (24), Carbone and Kohn (25), and Liu et al. (26) for ITS, TEF 1-α, and 5F2/7CR, respectively. The PCR products were analyzed via 1.5% agarose gel electrophoresis, visualized, and photographed under UV light. The phylogeny of six FIESC isolates obtained in this study, along with 33 reference sequences from GenBank (Table 1), was analyzed using multi-locus sequence typing (MLST). Sequences were assembled using the BioEdit Sequence Alignment Editor (27) and aligned using Clustal W. Minor gaps were manually removed using Molecular Evolutionary Genetics Analysis software version 7.0 (MEGA 7.0). Phylogenetic analysis was performed using the maximum likelihood method with 1,000 bootstrap replicates.

### Morphological characteristic

A single-spore culture was used to examine the morphological characteristics of FIESC isolates. Colony pigmentation was assessed after culturing the isolates on Potato Dextrose Agar (PDA) plates and incubating at 28°C for one week. Approximately 30 individual conidia were measured for length, width, and number of septa in microconidia and macroconidia, cultured on Spezieller Nahrstoffarmer Agar (SNA) medium, and incubated in the dark at 28°C for one week. Conidial characteristics were analyzed from images captured using an Axioplan 2 imaging microscope (Zeiss, Germany).

### Inoculum preparation

Six *Fusarium* isolates collected from human, veterinary, and plant sources were used to investigate their ability to cross-infect, invade, and colonize human nails, porcine skin, and muskmelon fruit. Conidial suspensions were prepared as described by Namisy et al. (28). Briefly, a single spore from each isolate was cultured on PDA medium and incubated at 28°C for one week. The conidia were then washed once with sterile distilled water (SDW). Mycelia were removed by filtration through a Miracloth filter (MilliporeSigma, Burlington, MA, USA) to prepare the conidial suspension, and the concentration was adjusted to 10⁶ conidia/mL for all inoculation treatments.

### Inoculation assay

The *ex vivo* infection model was used to study the colonization of *Fusarium* isolates in human nails. Nails were collected from male and female volunteers, disinfected with 70% ethanol for 1 min, and rinsed three times with sterile distilled water (SDW). After drying on sterile paper under laminar airflow, the sterilized nails were soaked in an Eppendorf tube containing conidial suspension for 30 s. Mock controls were inoculated with SDW. The nails were then placed in sterile Petri dishes and incubated at 28°C for 4 days. *Fusarium* invasion in infected nails was assessed at 4 days post-inoculation (dpi) using an Axio Imager A1 microscope (Carl Zeiss AG, Jena, Germany) equipped with an X-Cite 120Q fluorescence illuminator system (Excelitas Technologies, USA) and an Axiocam 506 color camera. Nail sections were manually prepared, placed on glass slides, and stained with 10 µL of Calcofluor White solution (Sigma) and 10 µL of 10% potassium hydroxide solution to enhance resolution. The slides were incubated at room temperature in the dark for 1 min. Fluorescence expression of *Fusarium* isolates was visualized using a DAPI filter with excitation at 320–390 nm and emission at 430–490 nm. The experiment was performed in triplicate, repeated twice, and approximately 10 slices per replication were observed.

In the animal tissue inoculation assay, an *ex vivo* infection model using porcine skin was employed. Fresh skin samples were excised from euthanized animals, cut into small pieces (~2 cm²), disinfected with 70% ethanol for 5 min, and then rinsed three times with sterile distilled water (SDW) before drying under a biosafety cabinet. The samples were wounded using a sterile 24G needle, placed in Petri dishes, and inoculated with 20 µL of conidial suspension from each *Fusarium* isolate on the upper layer of the porcine skin epidermis. Mock controls were inoculated with SDW. The infected tissues and controls were incubated at 28°C for 4 days. The histological examination was performed according to standard protocols as described by Feldman and Wolfe (29). Infected tissues were fixed in 10% formaldehyde for 24 h, embedded in paraffin, sectioned into 3 μm thick slices using a microtome, and stained with hematoxylin and eosin (H&E). Fungal invasion in stained sections was observed using an Axio Imager A1 microscope (Carl Zeiss AG, Jena, Germany).

To investigate the colonization of *Fusarium* isolates in plants, healthy muskmelon fruits were surface-disinfected with 70% ethanol for 30 s, followed by 1% NaOCl for 5 min, and then rinsed three times with sterile distilled water (SDW). The fruits were wounded using a sterile needle and inoculated with 10 µL (concentration 10^6^ conidia/mL) of conidial suspension, while mock controls were inoculated with SDW. The fruits were monitored for symptoms 10 days after incubation at 28°C.

### Statistical analysis

Data analyses were conducted using the R Statistical Software v4.1.3 (https://www.r-project.org/). To analyze the statistical significance of penetration depth of each *Fusarium* isolate, we performed a one-way analysis of variance followed by Fisher’s least significant difference multiple comparisons test at (*P* < 0.05).

## RESULTS

### Phylogenetic analysis

**Fig 1:**
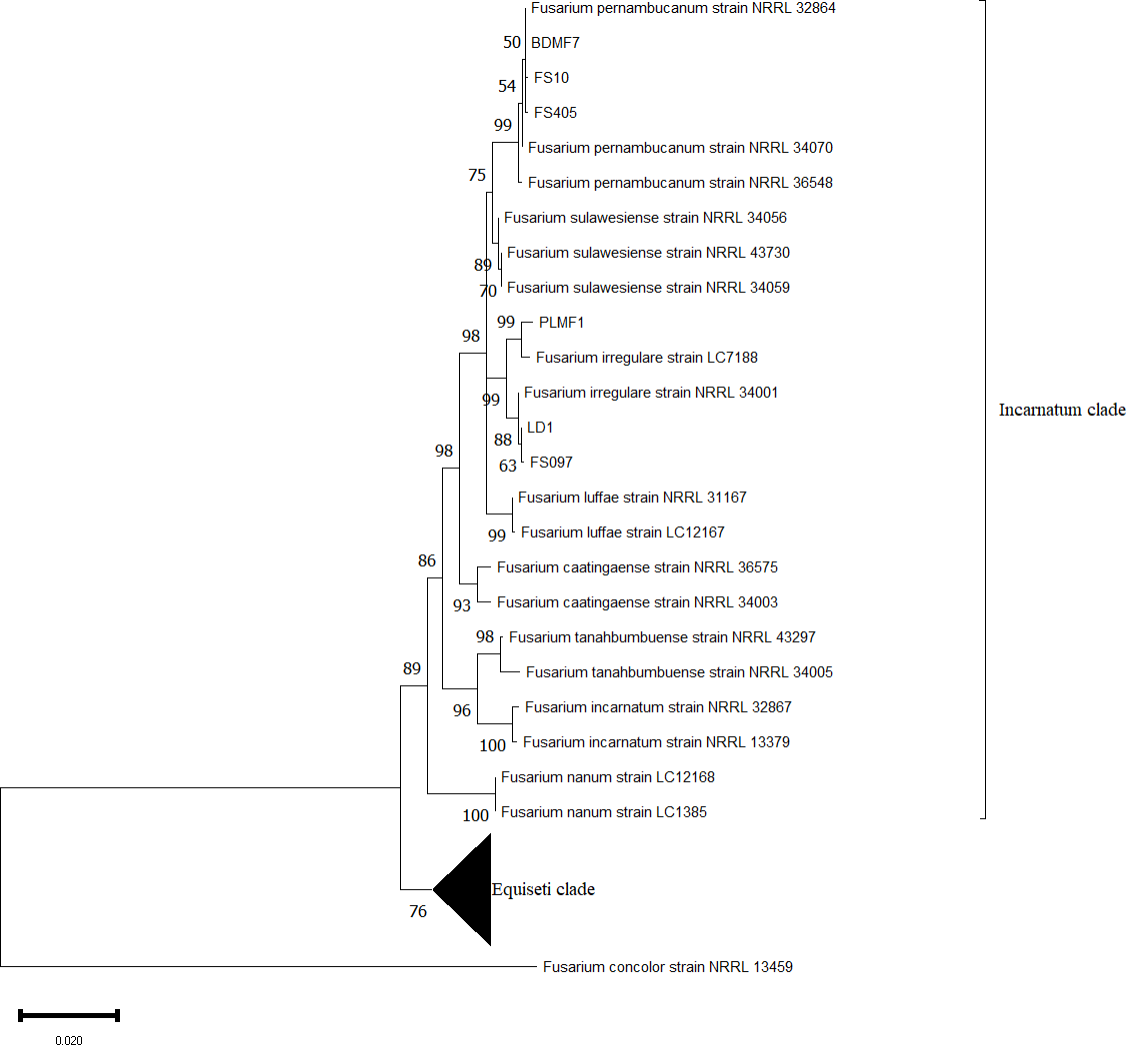
Phylogenetic tree of *Fusarium incarnatum-equiseti* species complex (FIESC) based on sequencing analysis of ITS, TEF 1-α, and RPB2 using the maximum likelihood method. The percentage of replicate trees in which the associated taxa clustered together in the bootstrap test (1,000 replicates) is shown.

The phylogenetic analysis was conducted based on the combined sequences of the ITS region, TEF 1-α, and RPB2 to classify six *Fusarium* isolates belonging to the *Fusarium incarnatum-equiseti* species complex (FIESC), which were isolated from human, veterinary, and plant sources. These sequences were compared with 33 reference sequences from GenBank, with *Fusarium* concolor (NRRL 13459) used as the outgroup (Table 1). The results indicated that the six isolates in this study were divided into two groups. Two isolates, LD1 and FS097, obtained from veterinary sources, and one isolate PLMF1 from a plant source clustered with *F. irregulare*, with 99% maximum likelihood (ML) bootstrap support. Similarly, two isolates, FS405 and FS10, obtained from veterinary and human sources, respectively, along with one isolate BDMF7 from a plant source, clustered with *F. pernambucanum*, also supported by 99% mL bootstrap values (Fig. 1).

### Morphological characteristic

**Fig 2:**
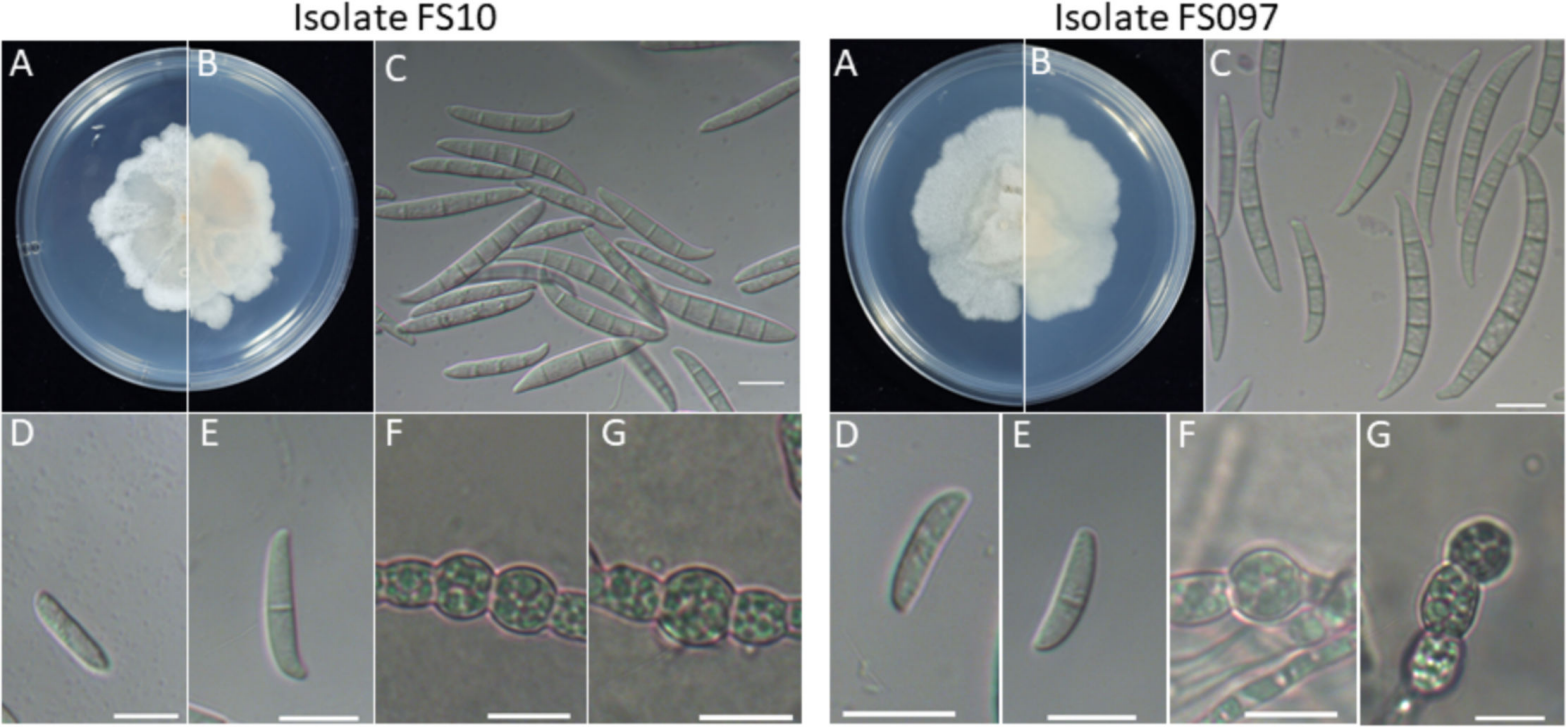
General morphology of *Fusarium pernambucanum* human isolate FS10 and *Fusarium irregulare* veterinary isolate FS097. (**A, B**) Forward and reverse colony morphology on PDA medium; (**C**) macroconidia; (**D, E**) microconidia; and (**F, G**) chlamydospores. Scale bar: 10 µm.

To further investigate their morphological characteristics, the FS097 and FS10 were selected as representative isolates and compared their morphology with same species of PLMF1 and BDMF7, isolates obtained from plant sources. Single-spore isolates assessed morphological characteristics, including the shape, size, and septation of microconidia and macroconidia. The colony of *F. irregulare* veterinary isolate FS097 exhibited cottony white mycelium, which developed white-brown pigmentation over time. Microconidia were ovoid to fusiform, or slightly curved, with 0 to 1 septum, measuring 2.4–3.7 µm × 10.1–16.5 µm. Macroconidia were 3 to 6-septate, with a slightly curved apical cell that ended bluntly, ranging in size from 3.2 to 4.8 µm × 24.6–43.0 µm. Spherical or oval chlamydospores with thick walls were abundant, occurring singly or in chains and produced in a terminal or intercalary position. These chlamydospores were developed through the modification of hyphal and conidial cells (Fig. 2).

**Fig 3:**
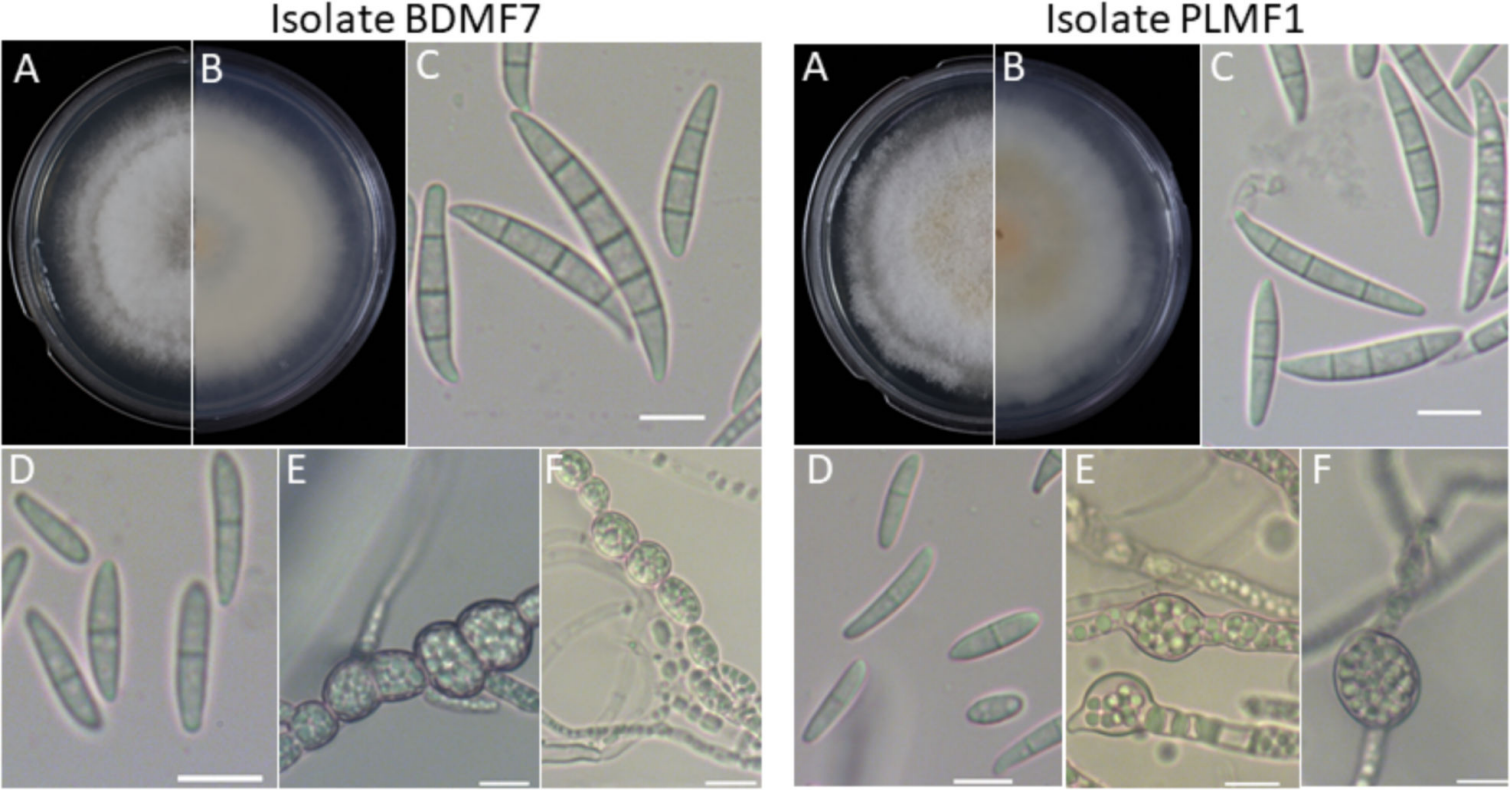
General morphology of *Fusarium pernambucanum* plant isolate BDMF7 and *Fusarium irregulare* plant isolate PLMF1. (**A, B**) Forward and reverse colony morphology on PDA medium; (**C**) macroconidia; (**D**) microconidia; and (**E, F**) chlamydospores. Scale bar: 10 µm.

Additionally, the *F. irregulare* plant isolate PLMF1 formed a colony with a white cottony appearance and developed pale yellow pigmentation. Microconidia were ovoid or slightly curved, with 0 to 1 septum, ranging in size from 2.1 to 3.7 µm × 12.0–17.9 µm. Macroconidia were mostly 3-septate and rarely 5 and 6-septate, with a slightly curved apical cell that ended bluntly, measuring 3.4–4.8 µm × 25.2–38.3 µm. Oval chlamydospores were abundant, occurring singly and produced in either terminal or intercalary positions, developing from hyphae (Fig. 3).

Similarly, the colony of the *F. pernambucanum* human isolate FS10 exhibited similarities to the veterinary isolate FS097. Microconidia were ovoid or slightly curved, with 0 to 1 septum, ranging in size from 2.0 to 3.6 µm × 11.5–16.8 µm. Most macroconidia were 3 to 5-septate, with rare occurrences of 7- and 11-septate. They featured a slightly curved apical cell that ended bluntly and measured 3.3–4.5 µm × 22.0–39.0 µm. Spherical chlamydospores occurred singly or in chains, produced in an intercalary position, and developed from hyphae (Fig. 2).

The colony of the *F. pernambucanum* plant isolate BDMF7 was cottony white with pale yellow pigmentation. Microconidia were ovoid or slightly curved, with 0 to 1 septum, ranging in size from 2.0 to 3.1 µm × 11.0–16.9 µm. Macroconidia were primarily 3-septate, with a few exhibiting 5 septa. The apical cell was blunt at the apex and measured 3.5–5.5 µm × 22.3–37.0 µm. Spherical chlamydospores were developed from hyphae and produced singly in terminal or intercalary positions (Fig. 3).

### Inoculation assay

In this study, we evaluated the potential for cross-infection in human nails, porcine skin tissue, and muskmelon fruits using *F. irregulare* and *F. pernambucanum* isolates obtained from various sources, including the human isolate FS10; veterinary isolates FS097, LD1, and FS405; and plant isolates PLMF1 and BDMF7.

**Fig 4:**
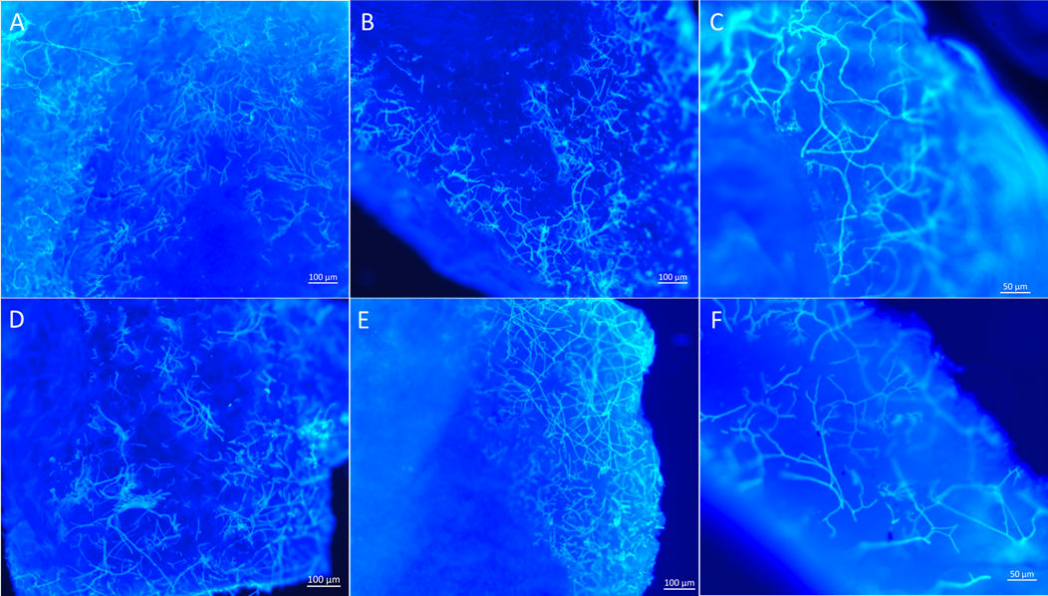
Histopathological characteristics of *Fusarium* isolates in human nail at 4 dpi. (**A**) *Fusarium pernambucanum* human isolate FS10; (**B**) *Fusarium pernambucanum* veterinary isolate FS405; (**C**) *Fusarium irregulare* veterinary isolate FS097; (**D**) *Fusarium irregulare* veterinary isolate LD1; (**E**) *Fusarium irregulare* plant isolate PLMF1; and (**F**) *Fusarium pernambucanum* plant isolate BDMF7.

**Fig 5:**
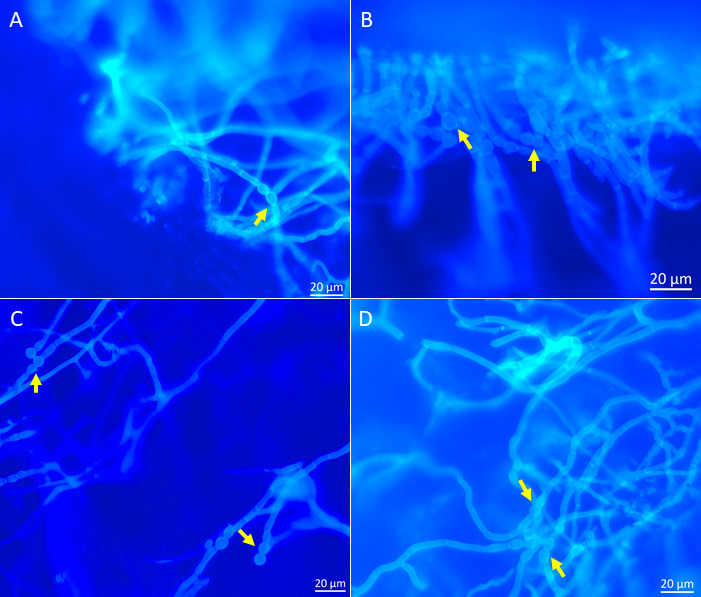
Chlamydospores in the human nail infected with *Fusarium* isolates at 4 dpi. (**A**) *Fusarium irregulare* veterinary isolate FS097; (**B**) *Fusarium pernambucanum* veterinary isolate FS405; (**C**) *Fusarium pernambucanum* plant isolate BDMF7; and (**D**) *Fusarium irregulare* plant isolate PLMF1.

The nail infection assay results demonstrated that all *Fusarium* isolates could grow on the nail surface within 4 days of incubation at 28°C. Additionally, histological analysis confirmed that all isolates could invade the nail plate (Fig. 4). However, the penetration rate varied among the different *Fusarium* isolates. The human and veterinary isolates exhibited a higher penetration density at 4 days post-inoculation compared to the plant isolates. Thick hyphae and chlamydospores were observed throughout the nail (Fig. 5). However, microconidia and macroconidia were not detected at 4 days post-inoculation.

**Fig 6:**
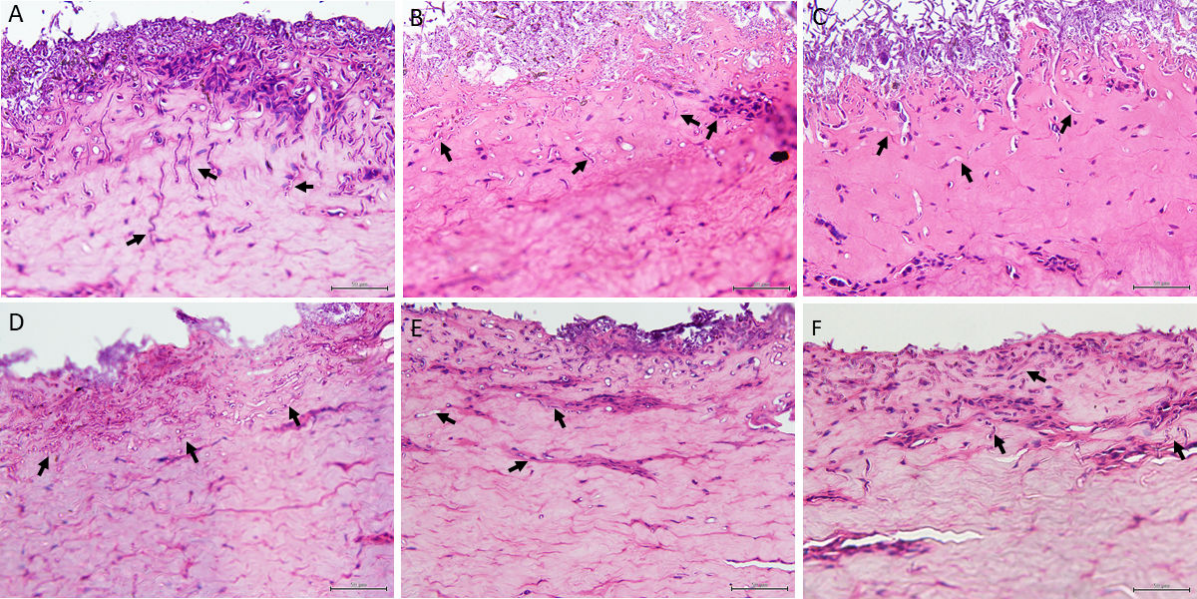
Histopathological characteristics of *Fusarium* isolates in porcine skin at 4 dpi. (**A**) *Fusarium pernambucanum* human isolate FS10; (**B**) *Fusarium pernambucanum* veterinary isolate FS405; (**C**) *Fusarium irregulare* veterinary isolate FS097; (**D**) *Fusarium irregulare* veterinary isolate LD1; (**E**) *Fusarium irregulare* plant isolate PLMF1; and (**F**) *Fusarium pernambucanum* plant isolate BDMF7.

In the porcine skin infection assay, an *ex vivo* infection model using porcine skin was employed to study the invasion and colonization of *Fusarium* isolates. The results indicated that all *Fusarium* isolates could colonize the surface of tissue samples within 2 days post-inoculation. The human isolate FS10 and veterinary isolates FS097, LD1, and FS405 exhibited abundant growth, whereas the plant isolates BDMF1 and PLMF1 showed lower growth, with BDMF1 displaying the least. Additionally, at 2 days post-inoculation, histological analysis confirmed that the pathogen could penetrate the epidermis layer of porcine skin. By 4 days post-inoculation, the penetration of all isolates had extended into the dermis layer (Fig. 6).

However, significant differences in penetration depth were observed among the *Fusarium* isolates. The human isolate FS10 displayed the most significant penetration depth, measuring 223.77 µm, followed by the veterinary isolates FS405 (191.43 µm), FS097 (190.65 µm), and LD1 (146.86 µm) (Table 2).

**TABLE 2:** Penetration depth of *Fusarium incarnatum-equiseti* species complex isolates in porcine skin and muskmelon fruit at 4 and 10 dpi, respectively

			Penetration depth	
Isolates	*Fusarium* species	Host	Porcine skin (µm)^*a*^	Muskmelon fruit (mm)^*a*^
PLMF1	*F. irregulare*	Muskmelon fruit	109.5 ± 7.6 d	20.1 ± 0.4 a
LD1	*F. irregulare*	Cat	146.8 ± 2.8 c	17.7 ± 0.3 b
FS097	*F. irregulare*	Rabbit	190.6 ± 3.5 b	17.0 ± 0.6 b
FS405	*F. pernambucanum*	Turtle	191.4 ± 5.1 b	20.8 ± 0.4 a
FS10	*F. pernambucanum*	Human	223.7 ± 4.8 a	18.4 ± 0.6 b
BDMF7	*F. pernambucanum*	Muskmelon fruit	84.90 ± 2.3 e	21.5 ± 0.2 a

^*a*^ Different letters in the same column indicate significant differences according to Fisher’s protected least significant difference test (*P* = 0.05).

**Fig 7:**
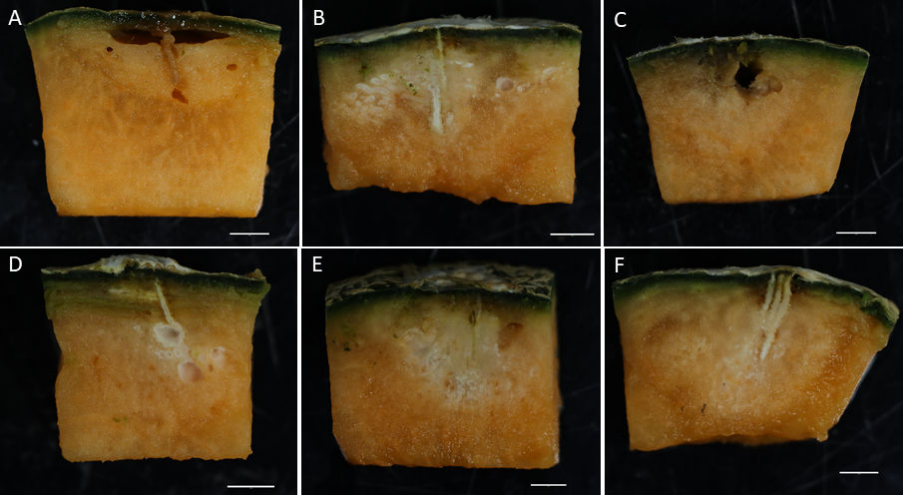
Invasion and colonization of *Fusarium* isolates in muskmelon fruit at 10 dpi. (**A**) *Fusarium pernambucanum* human isolate FS10; (**B**) *Fusarium pernambucanum* veterinary isolate FS405; (**C**) *Fusarium irregulare* veterinary isolate FS097; (**D**) *Fusarium irregulare* veterinary isolate LD1; (**E**) *Fusarium irregulare* plant isolate PLMF1; and (**F**) *Fusarium pernambucanum* plant isolate BDMF7.

Similar to infections observed in human nails and porcine skin, the plant pathogenicity assay also confirmed that all *Fusarium* isolates were pathogenic to muskmelon fruits at 10 days post-inoculation (dpi) (Fig. 7). Symptoms initially appeared as water-soaked lesions at the inoculation site. Over time, white, cotton-like mycelia developed on the fruit’s surface, progressively expanding and invading internal tissues, ultimately leading to discoloration and fruit breakdown. Additionally, the pathogen spread and colonized seeds within infected fruits. Pathogens were successfully reisolated from infected fruit and confirmed through morphological and molecular analysis. Among the isolates, *F. pernambucanum* plant isolates BDMF7 and veterinary isolate FS405 exhibited the greatest penetration depths, measuring 21.58 mm and 20.85 mm, respectively. Similarly, the *F. irregulare* plant isolate PLMF1 showed a significant penetration depth of 20.1 mm. In contrast, the *F. pernambucanum* human isolate FS10 and *F. irregulare* veterinary isolates LD1 and FS097 demonstrated the lowest penetration depths, measuring 18.43 mm, 17.70 mm, and 17.00 mm, respectively (Table 2).

## DISCUSSION

In this study, we identified FIESC as a pathogenic fungus capable of causing cross-infection in various hosts, including humans, animals, and plants. Notably, this pathogen was first reported in Taiwan in 2023, causing severe fruit rot in muskmelon fruit in different locations (30). Simultaneously, we successfully isolated the pathogen from human and veterinary sources. In humans, the pathogen was isolated from the sputum of a patient diagnosed with fungal pneumonia, who had a history of liver transplantation. *Fusarium* species have been reported to cause pneumonia, particularly in immunocompromised patients (10). Often manifesting as nodular and cavitary lesions, this condition is associated with increased mortality, even after controlling for immune status (31). Additionally, the pathogen has been isolated from a rabbit’s purulent nasal discharge, cat lung, and turtle. Similarly, *Fusarium* has been identified in the skin, nasal cavity, and lungs of Boer goats (13). In this study, the isolation of FIESC from diverse hosts and locations suggests that this fungus is widespread throughout Taiwan and capable of cross-infection across different species.

The commonly sequenced genes used for *Fusarium* identification include translation elongation factor-1α (TEF-1α), RNA polymerase 1 and 2 (RPB1 and RPB2), and β-tubulin (tub) (1). In this study, we used a combination of sequences of three genes, ITS, TEF 1-α, and RPB2, for identifying the FIESC isolates from plant, veterinary, and human sources. Based on the MLST results, all isolates belonging to the Incarnatum clade include two veterinary isolates, FS097 and LD1, and plant isolate PLMF1 which clustered with *F. irregulare*. Meanwhile, the veterinary isolate FS405, human isolate FS10, and plant isolate BDMF7 are clustered with *F. pernambucanum*. Previous multi-locus phylogenetic analyses identified 32 phylogenetic species within FIESC, which are divided into two major clades: the Equiseti clade and the Incarnatum clade (32, 33).

We examined the morphological characteristics of *Fusarium* isolates obtained from various host sources within the same species and incubated at 28°C, which has been reported as the optimal condition for observing FIESC isolate (30). Our findings revealed that differences among these isolates were observed in colony morphology and growth rate. Additionally, our results revealed that all *F. irregulare* and *F. pernambucanum* isolates from different hosts could produce an abundance of microconidia and chlamydospores on potato dextrose agar (PDA). However, in contrast to our findings, microconidia and chlamydospores were not observed in *F. irregulare* isolated from bamboo on PDA media (22). In *F. pernambucanum* isolate from humans, the predominant macroconidia exhibited 3 to 5 septa, whereas in plant isolate, most exhibited 3 septa, with a few displaying 4 or 5 septa. Both plant and human isolates demonstrated the ability to produce microconidia and chlamydospores on PDA. Additionally, Zhang et al. (34) reported that macroconidia of *F. pernambucanum* isolated from muskmelon had 3 to 5 septa on carnation leaf agar (CLA); however, no microconidia were observed. These morphological variations between our results and previous studies may be attributable to differences in cultural conditions. Various factors, including culture media, pH, and temperature, can significantly influence the mycelial growth and sporulation of *Fusarium* (35). Additionally, *Fusarium* isolates exhibit considerable variability in phenotypic characteristics, including colony color, pigmentation, sporulation, conidial size, and shape, even for the same species isolates (36). In this study, the morphological characteristics appeared to correlate more closely with host origin, which may indicate a degree of phenotypic plasticity. However, further investigation involving a larger number of isolates is necessary to validate these findings and assess the extent of host-driven morphological variation.

In this study, we examined the ability of *Fusarium* isolates obtained from various sources to penetrate and colonize the human nail plate, porcine skin, and muskmelon fruits. For all inoculation treatments, the spore suspension was standardized to a single concentration of 10⁶ conidia/mL, and SDW was used as a mock control due to the difficulty in identifying and sourcing a suitable negative control species. Notably, a previous study employed a single inoculum concentration to evaluate the pathogenicity of *Fusarium* species in plants and animals (30, 37). In the human nail infection test, results revealed that *F. pernambucanum* and *F. irregulare* isolates, regardless of their source, exhibited a remarkable ability to penetrate and colonize the nail plate within 4 days post-inoculation (dpi). This finding highlights their potential to cause onychomycosis in humans and underscores the adaptability of *Fusarium* across various environments and host types. Onychomycosis is the most common chronic fungal infection of the nail, accounting for approximately 50% of all nail diseases (38, 39). The most common fungi causing onychomycosis worldwide include *Fusarium* species, *Aspergillus* species, *Scopulariopsis brevicaulis*, *Neoscytalidium dimidiatum*, and *Acremonium* species (40).

Microscopic examination of *Fusarium* isolates colonized in the nail revealed that all isolates could produce chlamydospores at 4 dpi, indicating that the *Fusarium* isolates could establish an infection in a short time. However, no micro- or macroconidia were observed in infected nails. Previous studies reported the presence of chlamydospores and microconidia of *Fusarium* spp. throughout infected nails (41, 42) and in the reticular dermis, particularly associated with dermal blood vessels (43). The formation of chlamydospores in infected nails may aid in distinguishing dermatophytes from non-dermatophyte fungal causes of onychomycosis. Unlike previous studies, we observed no microconidia in infected nails. This discrepancy may be attributed to environmental factors or the specific *Fusarium* isolate examined.

Porcine skin was used as an *ex vivo* model to examine the ability of *Fusarium* isolates to infect human and animal skin. This model is frequently utilized due to its structural similarities to human skin, including thickness, hair follicle content, pigmentation, collagen, and lipid composition (44, 45). The results demonstrated that all *Fusarium* isolates could penetrate the dermal layer within 4 days post-inoculation. These findings suggest that FIESC isolates from various sources, including plant isolates PLMF1 and BDMF7, may cause both superficial and invasive diseases in humans and animals. This highlights *Fusarium*’s ability to transition from a plant pathogen to a zoonotic pathogen, underscoring its significance as a concern for ecosystems and public health. Similarly, Tava et al. (46) reported that strains of *F. musae* isolated from banana fruits and human patients can cause significant diseases in the banana as a plant host and *Galleria mellonella* larvae as a human proxy for the investigation of cross-kingdom pathogenicity. However, the severity of *Fusarium* infections in humans and animals is influenced by factors such as the *Fusarium* species and the host’s immune competence, with immunocompromised individuals being particularly susceptible to severe disease (31).

Beyond causing onychomycosis, *Fusarium* species can lead to invasive infections in humans, affecting the skin, lungs, blood vessels, and sinuses, often resulting in positive blood cultures (10). The skin is the most frequently affected organ in invasive infection, either as the primary site or through hematogenous spread, serving as an essential and early diagnostic clue for cutaneous lesions (10, 47). In addition to human infection, *Fusarium* has been reported to induce cutaneous, nasal, and systemic disease in a goat; the systemic infection was characterized by multifocal nodular lesions throughout the kidneys, abdominal lymph nodes, and lungs (13). Furthermore, *Fusarium* spp. have been associated with meningoencephalitis and chronic ulcerative dermatitis in dogs (11, 14).

Although all *Fusarium* isolates were capable of penetrating the dermal layer of porcine skin, our results indicated significant differences in penetration depth among isolates from various sources, including those within a single species. For instance, the human isolate FS10, which belongs to *F. pernambucanum*, exhibited a high penetration level, whereas the plant isolate BDMF7, also from the same species, demonstrated significantly lower penetration. These differences in penetration depth may reflect variations in pathogenicity. Our findings suggest that the pathogenicity of *Fusarium* isolates against porcine skin may depend more on their source than on their species. In contrast, a study examining the pathogenicity of *Fusarium* spp. isolates from clinical and environmental sources in a murine model found that virulence varied by species, with isolates belonging to *F. solani* being the most virulent (37). Additionally, virulence variations of *F. musae* strains in plant and animal hosts appear to depend more on the specific strain than on the host species (46). Based on our results and previous studies, the variance of *Fusarium* may be correlated to both the specific species and the individual isolate within that species. Furthermore, environmental conditions, such as temperature and humidity, can also influence the pathogenicity of *Fusarium* (48).

FIESC isolates from human, animal, and plant sources were evaluated for their pathogenicity against muskmelon fruits. The results indicated that all FIESC isolates were pathogenic to muskmelon, exhibiting different penetration depths at 10 days post-inoculation. FIESC has been reported to cause severe fruit rot disease in muskmelon in Taiwan (30). Furthermore, FIESC has been documented across various geographic regions as a causative agent of diseases in many crops, including tomato, eggplant, rockmelon, maize, soybean, rice, wheat, and barley (49–55). After inoculation with FIESC isolates from different sources, muskmelon fruits developed symptoms consistent with those observed on melon fruits under field conditions and as documented in previous reports (30, 56). Beyond its agricultural effects, FIESC poses a health risk to humans and animals. Transmission can occur through contaminated food, leading to either superficial or invasive infections. Additionally, these pathogens pose an indirect health hazard by producing a variety of mycotoxins (57). Currently, the pathogen has been reported in multiple regions, including Asia, Europe, and Africa, highlighting its global significance (58–60). Its wide distribution emphasizes the potential for cross-infection among plants, animals, and humans, as well as the associated risks of mycotoxin contamination.

### Conclusion

In this study, we report that *Fusarium* isolates belonging to *F. pernambucanum* and *F. irregulare* obtained from various sources, including human, cat, rabbit, and turtle, along with plant samples from muskmelon fruits, represent newly identified pathogens in Taiwan. These isolates exhibit potential for cross-infection among plants, animals, and humans, highlighting their significance in both agricultural and medical contexts. Notably, our findings demonstrate the possibility of transmitting from a plant pathogen to a zoonotic pathogen of the plant isolates PLMF1 and BDMF7. To our knowledge, this is the first report of *F. incarnatum* cross-infection among humans, animals, and plants. The ability of this pathogen to adapt across hosts underscores the urgent need for comprehensive strategies to mitigate its impact on ecosystems and human health. In future research, we plan to investigate the mechanisms of cross-infection to better understand how fungal pathogens transition between biological systems. Additionally, it will be essential to evaluate their ability to produce mycotoxins within human and animal tissues to assess potential health risks.

## Data Availability

All data generated or analyzed during this study are included in this published article.

## SUPPLEMENTAL MATERIAL

Table S1 spectrum.01919-25-s0001.docx Morphological characteristics of Fusarium incarnatum-equiseti species complex isolates used in this study.

ASM does not own the copyrights to Supplemental Material that may be linked to, or accessed through, an article. The authors have granted ASM a non-exclusive, world-wide license to publish the Supplemental Material files. Please contact the corresponding author directly for reuse.
