# Supplementary material for: Fusarium incarnatum: a paradigm for One Health pathogen dynamics across humans, animals, and the environment
Source: Microbiol Spectr. 2025 Nov 26;14(1):e01919-25. doi: 10.1128/spectrum.01919-25 (PMC12772272; doi:10.1128/spectrum.01919-25)
Supplement: Table S1 — Morphological characteristics of Fusarium incarnatum-equiseti species complex isolates used in this study. [file spectrum.01919-25-s0001.docx]

**Supplemental Table S1.** Morphological characteristics of *Fusarium* *incarnatum-equiseti* species complex isolates used in this study

| Isolates | Colony |  | Macroconidia | | | |  | Microconidia | | | |  |  |
| --- | --- | --- | --- | --- | --- | --- | --- | --- | --- | --- | --- | --- | --- |
|  |  |  | Shape | Wide (μm) | Length (μm) | No. of septa |  | Shape | Wide (μm) | Length (μm) | No. of septa |  | chlamydospore |
| PLMF1 | Cottony, white- yellow |  | Falcate, slightly curved | 3.4–4.8 | 25.2–38.3 | 3-5 |  | Ovoid to fusiform, slightly curved | 2.1–3.7 | 12.0–17.9 | 0-1 |  | single |
| LD1 | Cottony, white |  | Falcate, slightly curved | 3.3-4.7 | 26.7-36.6 | 3-5 |  | Ovoid to fusiform, slightly curved | 2.4-3.6 | 10.6-15.5 | 0-1 |  | single or chain |
| FS097 | Cottony, white-brown |  | Falcate, slightly curved | 3.2–4.8 | 24.6–43.0 | 3-6 |  | Ovoid to fusiform, slightly curved | 2.4–3.7 | 10.1–16.5 | 0-1 |  | single or chain |
| FS405 | Cottony, white- yellow |  | Falcate, slightly curved | 3.5-5.0 | 26.3-36.5 | 3-5 |  | Ovoid to fusiform, slightly curved | 2.3-3.6 | 11.4-16.3 | 0-1 |  | single or chain |
| FS10 | Cottony, white- brown |  | Falcate, slightly curved | 3.3–4.5 | 22.0–39.0 | 3-5 |  | Ovoid to fusiform, slightly curved | 2.0–3.6 | 11.5–16.8 | 0-1 |  | single or chain |
| BDMF7 | Cottony, white-yellow |  | Falcate, slightly curved | 3.5–5.5 | 22.3–37.0 | 3-5 |  | Ovoid to fusiform, slightly curved | 2.0–3.1 | 11.0–16.9 | 0-1 |  | single or chain |
